# Malpositioning of Prosthesis: Patient-specific Total Knee Arthroplasty Versus Standard Off-the-Shelf Total Knee Arthroplasty

**DOI:** 10.5435/JAAOSGlobal-D-17-00020

**Published:** 2017-08-02

**Authors:** Kyoung-Tak Kang, Juhyun Son, Oh-Ryong Kwon, Yong-Gon Koh

**Affiliations:** Department of Mechanical Engineering (Dr. Kang and Mr. Son), Yonsei University, Seoul, Republic of Korea, and the Department of Orthopaedic Surgery (Dr. Kwon and Dr. Koh), Joint Reconstruction Center, Yonsei Sarang Hospital, Seoul.

## Abstract

**Introduction:**

A recent study has challenged the premise that a patient-specific (PS)–designed total knee arthroplasty (TKA) component has better clinical survival than an off-the-shelf (OTS) prosthesis.

**Methods:**

We developed the finite element models for PS TKA and OTS TKA with 5° varus and valgus malalignment and 5° internal and external malrotations.

**Results:**

Contact stress on the medial side of the insert increased with internal femoral malrotation and varus tibial malalignment, but it decreased with external femoral malrotation and varus tibial malalignment in both PS TKA and OTS TKA. An increase in ligament force occurred in valgus malalignment and external malrotation, and in particular, the force exerted on the medial collateral ligament increased. However, PS TKA provided better biomechanical effects than did the standard OTS TKA with malpositioning in TKA.

**Discussion:**

These results emphasize the importance of precise surgical preservation in regard to the TKA position.

The importance of correct component position alignment in total knee arthroplasty (TKA) is well established. Various failure mechanisms after TKA have been noted, such as wear of a polyethylene (PE) insert, mechanical instability, and aseptic loosening.^1,2,3^ The factors that mainly influence the success of TKA are the restoration of lower-extremity alignment, accurate position of implantation, and optimal gap balancing.^4^ Previous studies reported that the survival rate after TKA was improved, and the limb alignment had been restored in three cases of valgus and varus malalignment from the mechanical axis.^5^ Therefore, the purpose for surgeons to perform TKA is to achieve a postoperative limb alignment as close as the normal alignment and gap balancing in the knee joint. Alignment accuracy is determined by surgical technique precision, and computer-assisted surgery was developed to improve surgical accuracy for exclusion of outliers.^5,6,7^ Numerous computer-assisted surgery studies have evaluated the improvement in accuracy of coronal implant positioning in TKA.^8,9^ However, there has been no standard for precise postoperative anatomic knee alignment or malposition of femoral and tibial components to provide optimum prosthesis survival.^10^ Furthermore, a recent study reported that postoperative lower-extremity alignment is not correlated with the survival rate after TKA even at 15 years after surgery from a modern TKA.^11,12^ One of the potential causes is that improvement in implant designs reduces the risk of wear problems even with malposition of the component.^13^ The improvement in implant design could be based on flat-on-flat or curved-on-curved concepts between the femoral component and PE insert. Furthermore, it could be enhanced by design using the patient's own geometry based on the patient-specific (PS) technology. PS TKA with a customized femoral component, PE insert, and tibial baseplate is an alternative surgical treatment to standard off-the-shelf (OTS) TKA implant.^14,15^

Computational biomechanics analysis is a common method widely used in the assessment of prosthesis performance, but it is recommended for more qualitative comparison rather than a quantitative prediction in orthopaedic biomechanics. Kessler et al^16^ studied the effect of femoral component malrotation and reported that a rotating bearing had minimal effect in reducing the patellofemoral maltracking compared with a fixed bearing. Zumbrunn et al^17^ reported that biomimetic bicruciate–retaining TKA postoperatively resulted in an activity-dependent kinematics similar to healthy knees in vivo. However, to our knowledge, there has been no report evaluating the biomechanical effects of malpositioning of implantation with respect to PS TKA and standard OTS TKA.

Therefore, the purpose of this study was to determine the effect of malpositions with respect to 5° varus and valgus malalignments, and 5° internal and external malrotations under gait and deep knee bend loading conditions. The maximum contact stresses on the medial and lateral sides of the PE insert and the forces exerted on the collateral ligaments were evaluated. We hypothesized that PS TKA produces a more positive biomechanical effect on the knee joint over the standard OTS TKA in the implantation with malposition of the knee joint.

## Methods

### Normal Knee Model

**Figure 1:**
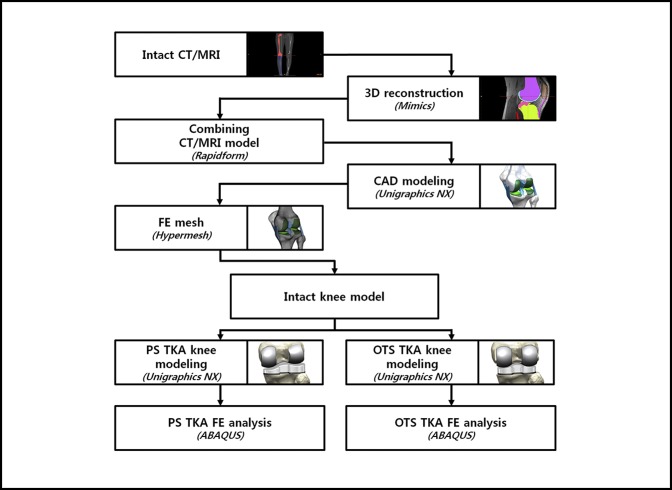
Development process for the final element (FE) model. CAD = computer-aided design, OTS = off the shelf, PS = patient-specific, TKA = total knee arthroplasty

A 3-dimensional (3D) nonlinear finite element (FE) model for the normal knee joint was developed using data from the CT and MRI scans of a healthy 36-year-old male subject. The development procedure for the FE model is described in Appendix 1. Figure 1 shows the development process for the FE model.

### Design of Patient-specific Total Knee Arthroplasty

The 3D reconstructed PS geometry of the anatomy and surface data were used to develop the geometry of PS TKA. In Mimics software, the 3D images were transformed to Standard Tessellation Language files and imported into the digital computer-aided design software 3-Matic (Materialise). 3-Matic version 9.0 (Materialise) enables the user to combine geometries from mixed sources into one project. The initial graphics exchange specification files exported from 3-Matic were imported into Unigraphics NX (version 7.0; Siemens PLM Software) to develop the PS TKA implant.

**Figure 2:**
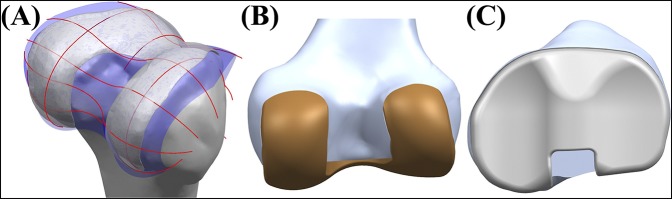
**A,** Surface geometry used in the PS TKA design. **B,** PS femoral component. **C,** PS PE insert with respect to the patient's bone geometry. PE = polyethylene, PS = patient-specific, TKA = total knee arthroplasty.

The sagittal geometry of the patient's bone was mainly used for the geometry of the PS femoral component. The three PS J-curves for the trochlear grooves and the medial and lateral condyles were generated in computer-aided design software with the reference of normal articular geometry of the subject in this study.^18,19,20^ Planes were extracted into the condyles in the sagittal view, in which the curves were used to duplicate the surface geometry articulations (Figure 2). In general, a femur of a patient in the coronal plane provides asymmetric lateral and medial condyles: the coronal offset. These individual differences were considered in the PS femoral component design. The coronal offset is understood as the difference in height between the medial and the lateral femoral condyles in the coronal extension plane, which may lead to an asymmetric extension gap that must be accounted for at the tibial articular surface and the posterior femoral condyles in the normal knee joint.^21^ Typically, the lateral posterior condyle is shorter than the medial condyle, which causes a unique asymmetric flexion gap.^21^ These femoral J-curves were matched with the PS PE inserts whose perimeters corresponded to each individual patient's tibial plateau, which preserved the distal medial-lateral offset of the patient's femoral condyles through the height of the PS PE insert that reflects the condylar offset with normal mechanical axis alignment.

### Finite Element Models for Patient-specific Total Knee Arthroplasty and Standard Off-the-Shelf Total Knee Arthroplasty

The standard OTS TKA FE model was developed using a 3D laser scanner, and the detailed procedures were described in a previous study.^22^ The standard OTS implant, Genesis II Total Knee System (Smith & Nephew), was used in this study.

**Figure 3:**
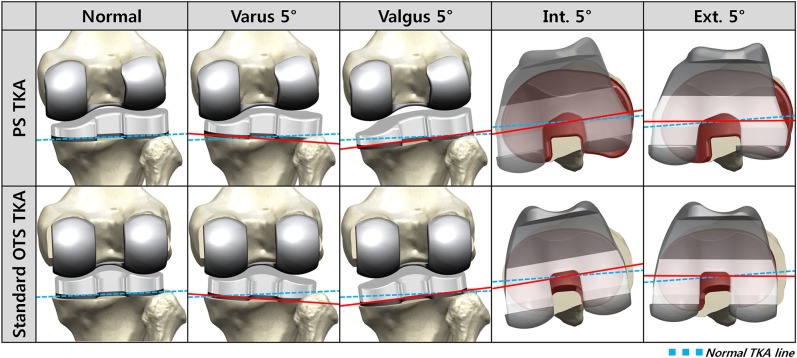
Finite element (FE) models used in this study. OTS = off the shelf, PS = patient-specific, TKA = total knee arthroplasty

Surgical simulation for TKA was performed by two experienced surgeons (O.-R.K. and Y.-G.K.). A neutral position FE model was developed for both PS and standard OTS TKAs using the normal mechanical axes and ligaments from the subject in this study (Figure 3). The neutral position FE model was developed according to the following surgical preferences: Default alignment for the femoral component rotation was parallel to the transepicondylar axis; the femoral component coronal alignment was perpendicular to the mechanical axis; or the femoral component sagittal alignment was at 3° flexion with a 9.5-mm distal medial resection. To develop the malpositioning TKA models, four different cases were considered with respect to the neutral position: 5° internal and external malrotations, and 5° varus and valgus malalignments. The tibial default alignment was rotated 0° to the AP axis; the coronal alignment was 90° to the mechanical axis; and the sagittal alignment was 5° of the posterior slope with an 8-mm resection below the highest point of the lateral plateau.

Contact conditions were considered in the interface between the femoral component and the PE insert and patellar button in TKA. The coefficient of friction between the PE and metal materials was assumed to be 0.04, which was consistently referred to the previous explicit FE models. The PE insert and patellar button were modeled with elastoplastic material properties.^23^ The materials for the femoral component, PE insert, and tibial baseplate were cobalt chromium (CoCr) alloy, ultra-high–molecular-weight PE, and titanium alloy (Ti6Al4V), respectively (Table 1). The femoral component and tibial baseplate in the models were fully bonded to the femur and tibia, respectively, representing bone cement application.

**Table 1:**
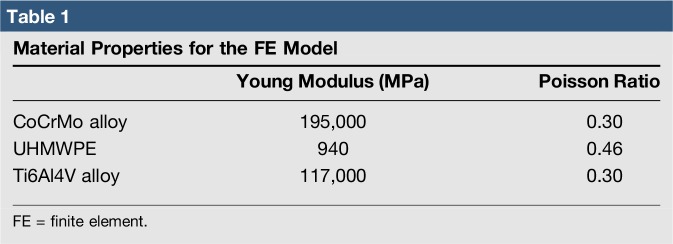
Material Properties for the FE Model

To evaluate the effect of PS TKA and standard OTS TKA with respect to malpositioning in TKA, the gait cycle loading conditions were applied to both the tibiofemoral and patellofemoral joint motions under deep knee bend loading conditions.^24,25^ The FE model was analyzed using ABAQUS software (version 6.11; Simulia). The results for the maximum contact stress on the PE insert were evaluated, and collateral ligament forces were evaluated in both PS TKA and standard OTS TKA surgical techniques.

## Results

### Effects of Patient-specific Total Knee Arthroplasty and Standard Off-the-Shelf Total Knee Arthroplasty on the Maximum Contact Stress for the Polyethylene Insert With Respect to Malpositioning in Total Knee Arthroplasty

**Figure 4:**
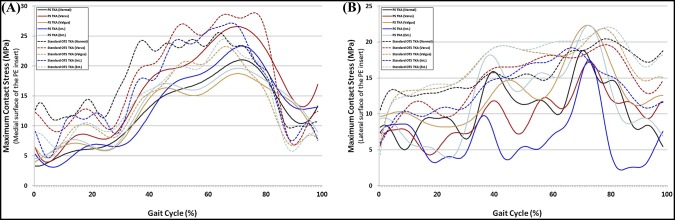
Comparison of maximum contact stress on the (**A**) medial surface and (**B**) lateral surface of the polyethylene (PE) insert with respect to malpositioning in total knee arthroplasty (TKA) during the gait cycle. OTS = off the shelf, PS = patient-specific

Figure 4 shows the maximum contact stress on the PE inserts in PS TKA and standard OTS TKA with respect to malpositioning in TKA during the gait cycle. In both PS TKA and standard OTS TKA surgical techniques, contact stress on the medial PE insert increased in varus tibial malalignment and internal femoral malrotation (Figure 4, A). Contrarily, contact stress on the lateral PE insert increased in valgus tibial malalignment and external femoral malrotation (Figure 4, B). The contact stress on the medial PE insert increased by 27% and 13%, respectively, in standard OTS TKA and PS TKA with varus tibial malalignment, whereas those increased by 11% and 6%, respectively, with internal femoral malrotation. The contact stress on the lateral PE insert increased by 19% and 7%, respectively, in standard OTS TKA and PS TKA with valgus tibial malalignment, whereas those increased by 19% and 8%, respectively, with external femoral malrotation.

**Figure 5:**
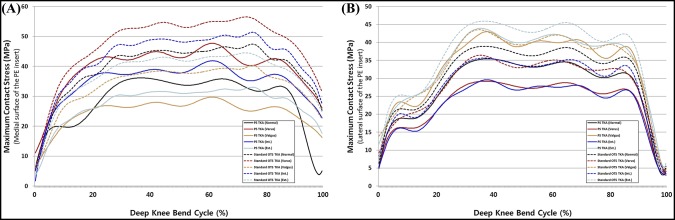
Comparison of maximum contact stress on the (**A**) medial surface and (**B**) lateral surface of the polyethylene (PE) insert with respect to malpositioning in total knee arthroplasty (TKA) during the deep knee bend cycle. OTS = off the shelf, PS = patient-specific

The maximum contact stresses on the PE inserts in PS TKA and standard OTS TKA are shown in Figure 5 with respect to malpositioning in TKA during deep knee bend loading conditions. There was a similar trend in deep knee bend with gait cycle condition in both PS and standard OTS TKA, and contact stress increase was even greater than gait cycle condition.

### Effects of Patient-specific Total Knee Arthroplasty and Standard Off-the-Shelf Total Knee Arthroplasty on the Collateral Ligament Forces With Respect to Malpositioning in Total Knee Arthroplasty

**Figure 6:**
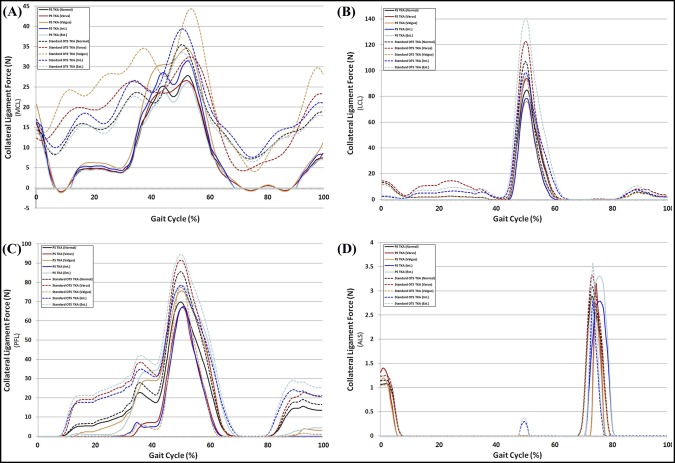
Comparison of ligament forces on the (**A**) MCL, (**B**) LCL, (**C**) PFL, and (**D**) ALS with respect to malpositioning in TKA during the gait cycle. ALS = anterior lateral structure, LCL = lateral collateral ligament, MCL = medial collateral ligament, OTS = off the shelf, PFL = popliteofibular ligament, PS = patient-specific, TKA = total knee arthroplasty

Figure 6 shows the ligament forces on the medial collateral ligament (MCL), lateral collateral ligament (LCL), popliteofibular ligament (PFL), and anterior lateral structure (ALS) in PS TKA and standard OTS TKA with respect to malpositioning in TKA during the gait cycle. The forces on the MCL increased in both PS TKA and standard OTS TKA with valgus and internal malalignment under gait loading conditions. The forces on the LCL, PFL, and ALS increased in both PS TKA and standard OTS TKA with varus malalignment and external malrotation under gait loading conditions.

**Figure 7:**
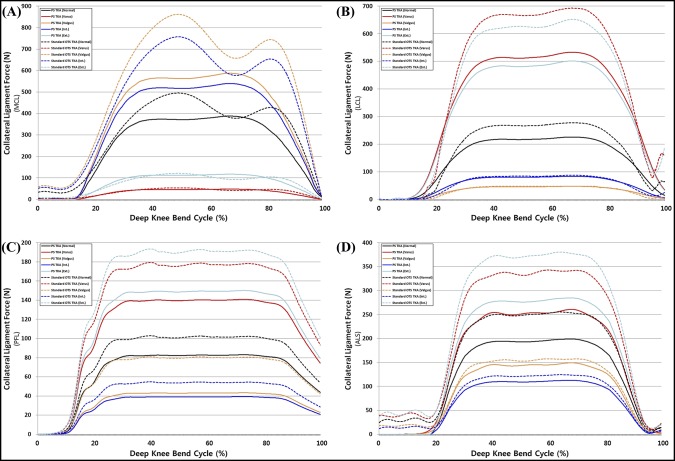
Comparison of ligament forces on the (**A**) MCL, (**B**) LCL, (**C**) PFL, and (**D**) ALS with respect to malpositioning in TKA during the deep knee bend cycle. ALS = anterior lateral structure, LCL = lateral collateral ligament, MCL = medial collateral ligament, OTS = off the shelf, PFL = popliteofibular ligament, PS = patient-specific, TKA = total knee arthroplasty

The ligament forces on the MCL, LCL, PFL, and ALS in PS TKA and standard OTS TKA with respect to malpositioning in TKA under deep knee bend loading conditions are shown in Figure 7. There was a similar trend in the deep knee bend with gait loading condition; however, the increase in forces exerted on ligaments was greater in the deep knee bend loading condition. The LCL, PFL, and ALS cases also increased in varus malalignment under both gait and deep knee bend loading conditions; however, the increase in forces was lower than that of valgus malalignment in both PS TKA and standard OTS TKA surgical techniques.

## Discussion

The most important finding of this study was that there were positive effects on the PE insert and the collateral ligaments in PS TKA compared with the standard OTS TKA with malpositioning of TKA. In particular, these positive effects were remarkably observed in the PE insert and the collateral ligaments under gait and deep knee bend loading conditions. Malposition of prosthesis implantation in TKA has been ascribed to several clinical complications. Despite the large variability in implantation positioning of the femoral and tibial components in TKA, the biomechanical effects of malposition on the knee joint functions have not yet been clearly understood and discovered.

The femoral malrotation in malpositioning in TKA is a common cause of revision surgery.^26^ Prosthesis implantation with precise rotation of the femoral component is critical for optimal patellofemoral tracking in the knee joint and for a generous flexion gap balancing. In addition, previous studies investigated the effect of lower-extremity alignment in TKA on the volumetric wear rate and found that there was a positive correlation between postoperative tibial varus alignment and wear of the PE insert in malpositioning with valgus malalignment.^27^ In malpositioning with valgus malalignment, previous studies reported that strain values from the valgus testing increased in both the anterior and posterior bands of the MCL, with the greatest average values found in the posterior band at 60° knee flexion.^28^

Therefore, we performed the computational simulation on the femoral malrotation and tibial malalignment in malpositioning cases that potentially causes a clinical problem after TKA. The advantage of a computational simulation with a single subject is that the effects of prosthesis alignment within the same subject exclude the variability in weight, height, bony geometry, material properties of ligaments, and component size.^29^ Contrarily, there is also a disadvantage that variability in differing ethnicity is not considered with a single subject.

Previous studies with computational simulation have often investigated separately malrotation and malalignment in TKA.^16,29,30^ Chen et al^31^ primarily performed computational simulation with malrotation and malalignment simultaneously, but it was limited to gait cycle loading condition. In addition, Nishikawa et al^13^ suggested that comprehensive factors of regarding conventional prostheses could improve the longevity of the PE insert and decrease the risk of surgical failures due to component malalignment. We suggest that the latest development in TKA surgical technology to be up to date is PS TKA.

We hypothesized that PS TKA reduces the negative effect on the PE insert with respect to contact stress and the collateral ligament forces, compared with the standard OTS in malpositioning implantation in TKA, because PS TKA considers the aspects of normal femoral condyles, such as the intercondylar notch distance, J-curve, condylar offset, AP and mediolateral widths, and the native tibial bone size and coverage. Furthermore, wider bone coverage with a PS design prosthesis can expand the contact area between the femoral component and PE insert, leading to less contact stress in TKA.

We found that contact stresses on medial and lateral PE inserts, respectively, increased in internal and external malrotations, and malalignments of varus in medial and valgus in lateral, in both PS and standard OTS TKA surgical techniques. Our findings regarding the maximum contact stress on the PE insert increasing in femoral malrotation and tibial malalignment were consistent with the results of previous studies for gait loading conditions.^31,32^ Our results also demonstrated that the effects from a 5° varus malrotation of the tibial component were slightly greater than those from the femoral malrotation.^31^ Varus tibial alignment that influences the stress distribution on the tibial component may lead to the increase of shear forces on the tibiofemoral interface, causing the higher risk of failure due to wear.^33^ The transverse stability of the knee during extension is mainly dependent on the collateral ligaments, and they become taut during extension and slacken during flexion.^34^ Femoral malrotation and tibial malalignment influenced the ligaments and the contact position on the PE insert, which directly contributed to the change of forces exerted on the collateral ligament with stress distribution, eventually leading to the changes in the predicted knee contact stress. We showed that the MCL force increased with internal malrotation and valgus malalignment. In addition, this trend was similar to the results of previous studies.^28,29,30^ In previous studies, the increased MCL tension was one of the principle causes of the pain or stiff knee in internal malrotation.^28,29,30^ Internal femoral component rotation and valgus tibial component malalignment may be detrimental to the MCL. For the LCL, PFL, and ALS, the ligament forces increased with external malrotation and varus malalignment. Our result showed that collateral ligament forces in internal malrotation and valgus malalignment were greater than those in external malrotation and varus malalignment. The increase in contact stress on the PE insert and collateral ligament forces in malpositioning on TKA was lower in PS TKA compared with a standard OTS TKA. The interesting finding was that the increase in ligament forces decreased in malpositioning in TKA. It was predictable for the decrease in contact stress in PS TKA, as contact area would have increased, but not for collateral ligament.

PS TKA was designed using patients' sagittal and coronal anatomic geometries, and kinematics of the knee joint could follow the kinematics in preoperative conditions even with malpositioning. In the native condition, stiffness of articular cartilage and the meniscus is remarkably lower than that of the PE insert, which means that those could be very flexible and easily transformed during dynamic activity, and they can be conserved using a PS TKA implant. Based on the results, the preservation of a normal mechanical axis, precise implant positioning, and optimal gap balancing are critical factors for successful TKA. However, PS TKA provided better biomechanical effects than did the standard OTS TKA with malpositioning in TKA, as it was designed with the patient's anatomic geometry.

There are several limitations to this study. First, this simulation was performed with a virtual and variable model, and the material properties of soft tissues used in this study referred to the relevant cadaveric studies. However, this methodology has been widely used in computational simulation studies.^29,31,34^ In addition, the previous study found that the trend was conserved even with the change in material properties.^29^ Second, the quadriceps and collateral ligaments may be released during operation for the better installation and stability in TKA, which could influence the functions and material properties of muscles and ligaments. However, the purpose of this study was to evaluate the effect of malpositioning on TKA without considering the effect of ligaments. Third, the results could not substitute clinical results and patient satisfaction because they are outcomes from FE analysis. However, contact stress on the PE insert and force exerted on ligaments are key factors that should be investigated for the evaluation of biomechanical effects in computational biomechanics.^20,29,30,31,35,36^ Fourth, elastoplastic material properties were used for the PE insert. An elastoplastic constitutive model does not provide time-dependent deformation with the stress lower than the yield stress used in the model. Substantial creep of the PE insert can occur in TKA, which may affect the contact areas and, therefore, the magnitude of the contact stresses. Finally, the standard OTS TKA used in this study could not represent all prostheses used in TKA because each design of prosthesis has a different design rationale.

In conclusion, the increase of forces on the MCL in valgus malalignment and internal malrotation may increase instability in TKA. The increase in contact stress on the PE insert in varus malalignment and femoral malrotation could lead to problems because of wear. However, the negative biomechanical effect in PS TKA was reduced over that of the standard OTS TKA with malpositioning in TKA. The FE modeling analysis has great potential to further improve TKA biomechanical outcome. These findings were revealed in computational models and need to be validated in a controlled clinical setting.

## Appendix 1 Procedure for FE model

CT and MRI were examined using a 64-channel CT scanner (Somatom Sensation 64; Siemens Healthcare) and a 3T MRI system (Discovery MR750w; GE Healthcare), respectively. The medical history of the subject was approved to have no musculoskeletal disorder and any related diseases arisen from malalignment in the lower extremity, which was considered to be a healthy knee joint. This computational knee joint model and its validation procedure were described in detail in previous studies.

To develop the 3D FE model, processing of 3D reconstruction was primarily performed. The CT and MRI images were segmented with software Mimics 17.0; Materialise for the 3D formation of lower-extremity structures; the combination of positional alignment from each FE model using Rapidform (3D Systems Korea Inc).

The bony structure was assumed to be rigid bodies. The articular cartilage and menisci were modeled as isotropic and transversely isotropic, respectively, with linear elastic material properties. In addition, the major ligaments were modeled with nonlinear and tension-only spring elements.

The force–displacement relationship based on the functional bundles in the actual ligament anatomy is shown in the following equation:

where *f (ε)* is the current force, *k* is the stiffness, *ε* is the strain, and *ε*_*1*_ is assumed to be constant at 0.03. The ligament bundle slack length *l*_*0*_ can be calculated by the reference bundle length *l*_*r*_ and the reference strain *ε*_*r*_ in the upright reference position.

The interfaces between the articular cartilage and the bones were assumed to be fully bonded. Six pairs of tibiofemoral contacts between the femoral cartilage and the meniscus, the meniscus and the tibial cartilage, and the femoral cartilage and the tibial cartilage were modeled for both the medial and lateral sides. A finite sliding frictionless hard contact algorithm with no penetration was applied to all contacts in all articulations.

Convergence was defined as a relative change of <5% between two adjacent meshes. The average element size of the simulated articular cartilage and menisci was 0.8 mm.
